# Epithelial‐to‐mesenchymal transition transcription factors in cancer‐associated fibroblasts

**DOI:** 10.1002/1878-0261.12080

**Published:** 2017-06-13

**Authors:** Josep Baulida

**Affiliations:** ^1^ Programa de Recerca en Càncer Institut Hospital del Mar d'Investigacions Mèdiques Barcelona Spain

**Keywords:** cancer‐associated fibroblasts, epithelial‐to‐mesenchymal transition transcription factors, Snail, tumour–stroma crosstalk, Twist, ZEB

## Abstract

Beyond inducing epithelial‐to‐mesenchymal transcription (EMT), transcriptional factors of the Snail, ZEB and Twist families (EMT‐TFs) control global plasticity programmes affecting cell stemness and fate. Literature addressing the reactivation of these factors in adult tumour cells is very extensive, as they enable cancer cell plasticity and fuel both tumour initiation and metastatic spread. Incipient data reveal that EMT‐TFs are also expressed in fibroblasts, providing these with additional properties. Here, I will review recent reports on the expression of EMT‐TFs in cancer‐associated fibroblasts (CAFs). The new model suggests that EMT‐TFs can be envisioned as essential metastasis and chemoresistance‐promoting molecules, thereby enabling coordinated plasticity programmes in parenchyma and stroma–tumour compartments.

**Abbreviations**
AKT2: serine/threonine kinase 2
ANLN: anillin actin binding protein
CAFs: cancer associated fibroblasts
CD44: CD44 antigen
CHK1: checkpoint kinase1
CXCL12: C‐X‐C motif chemokine ligand 12
DIAPH3: diaphanous related formin 3
E12/E47: E2A immunoglobulin enhancer‐binding factors 12 and E47
ECM: extracellular matrix
EMT: epithelial‐to‐mesenchymal transition
EMT‐TFs: EMT transcription factors
ERK2: extracellular signal‐regulated kinase 2
IL6: interleukin 6
LOX: lysyl oxidase
MCP‐3: monocyte chemotactic protein‐3
MSCs: mesenchymal stem cells
MYL9: myosin light chain 9
PDGF‐BB: platelet derived growth factor‐BB
PGE2: prostaglandin E2
PTEN: phosphatase and tensin homolog
ROCK: rho‐associated protein kinase
SCID: severe combined immunodeficient
SMAD: mothers against decapentaplegic homolog
STAT3: signal transducer and activator of transcription 3
TAZ: tafazzin
TGF‐b: transforming growth factor beta
USP7: ubiquitin‐specific peptidase 7
YAP1: yes associated protein 1
α‐SMA: alpha‐smooth muscle actin

## 1 Introduction

Ordinary fibroblasts orchestrate the organization and activities of the extracellular milieu, maintaining adult tissue homeostasis and contributing to proper cell communication and function. Their activity is exacerbated in restricted tissue areas by increased remodelling requirements, for instance in fibrotic tissues and in granulation tissues generated to heal epithelial wounds (Hinz, 2010). In agreement with their contractile ability, activated fibroblasts are named myofibroblasts. Similar fibroblasts, named cancer‐associated fibroblast or CAFs, are found around tumours, where they support biomechanical and biochemical remodelling of the stromal extracellular matrix that favours tumour malignance (Kuzet and Gaggioli, 2016; Malik *et al*., 2015).

In granulation and fibrotic tissues, TGF‐β and inflammatory molecules secreted by injured cells, or cells responding to their signals, promote a fibroblast–myofibroblast transition (Desmoulière *et al*., 1993). Similarly, molecules secreted by tumour cells support the emergence of CAFs (Rybinski *et al*., 2014; Webber *et al*., 2015) from resident fibroblasts and other host mesenchymal cells (Kuzet and Gaggioli, 2016; Polanska and Orimo, 2013). TGF‐β signalling is a key pathway for inducing CAF activity, tumour metastasis and malignance (Calon *et al*., 2014). Thus, in breast (Richardsen *et al*., 2012), colorectal (Calon *et al*., 2012; Tsushima *et al*., 1996) and prostate cancers (Steiner and Barrack, 1992), elevated expression of TGF‐β in the tumour area is associated with poor prognosis and locally advanced disease.

The Snail, ZEB and Twist proteins were initially described as EMT‐TFs. All of these factors are expressed in embryogenesis and regulate developmental programmes (Shook and Keller, 2003). Beyond EMT, we currently know that they are also involved in controlling global plasticity programmes affecting cell stemness and fate (Nieto and Cano, 2012), and aberrant reactivation of EMT‐TFs in adult tumour epithelial cells can promote cancer cell plasticity and trigger both tumour initiation and metastatic spread (Puisieux *et al*., 2014). In adult healthy tissues, EMT‐TFs are not expressed by the bulk of adult epithelial cells and fibroblasts. In contrast, a subset of mesodermal‐derived cells, mainly activated fibroblasts (Francí *et al*., 2006; Isenmann *et al*., 2009; Wang *et al*., 1997) (see below), were found to clearly express EMT‐TFs. This restricted expression suggests that EMT‐TFs provide fibroblasts with additional properties.

In contrast to the abundant information about the action of EMT‐TFs on epithelial cells, information about their actions in fibroblasts is just emerging. Here, I will review the incipient literature describing increased expression of EMT‐TF in CAFs. Available data suggest that EMT‐TFs are required for the action of CAF on extracellular paracrine and mechanical signalling that stimulate the expression of EMT‐TFs in adjacent tumour cells. Thus, EMT‐TFs promote coordinated cell plasticity changes in tumour parenchyma and stroma, supporting metastasis and chemoresistance.

## 2 Role of Snail proteins in fibroblasts

### 2.1 Snail1 expression in CAFs and other activated fibroblasts

Analysis of Snail1 expression in tissues has been hindered by the fact that the expression of Snail1 is tightly controlled at the post‐transcriptional level and usually cannot be correctly estimated by RNA analyses (Côme *et al*., 2006; Díaz and de Herreros, 2016; Díaz *et al*., 2014), and by the limitation of specific antibodies available. While commercial antibodies are not fully specific and give rise to abundant background, other antibodies require severe antigen retrieval conditions and intensive blocking and washing to obtain weak but specific staining. Francí *et al*. detected negligible reactivity in adult tissues and concluded that Snail1 was not constitutively expressed in most of the adult mesenchymal cells. In epithelial tumours, Snail1 expression presents a limited distribution restricted to stromal cells placed in the vicinity of the tumour and to tumour cells in the same areas (Francí *et al*., 2006). In specific types of breast tumours, Snail1 is also detected in both tumoural compartments (Côme *et al*., 2006).

A further analysis showed that expression of Snail1 protein in the stroma is a putative prognosis marker for colon tumours, as Snail1 immunoreactivity in this compartment was associated with the presence of metastasis and with lower specific survival of patients with cancer (Francí *et al*., 2009). These correlations were already detected in early tumour stages. In pharyngeal squamous cell carcinomas, Snail1 expression in tumour stromal myofibroblasts and endothelial cells also predicts poor survival (Jouppila‐Mättö *et al*., 2011b). Moreover, simultaneous expression of Snail1 and Twist1 in stromal cells is associated with clinical and histopathological characteristics that indicate disease progression, and negative expression of these EMT‐TFs predicts a better outcome (Jouppila‐Mättö *et al*., 2011a). The value of stromal Snail1 as a prognosis factor was further extended to infiltrating breast cancers, in which specimens with Snail1(+) CAF tend to exhibit desmoplastic areas with anisotropic fibres and are associated with lymph node involvement and poorer outcomes (Stanisavljevic *et al*., 2014).

Using a panel of cultured CAF lines established from colon cancer biopsies, Snail1 levels were shown to correlate with myofibroblast markers and activity on extracellular matrix (Stanisavljevic *et al*., 2014) and with their ability to promote tumour cell invasion in a paracrine manner (Herrera *et al*., 2013, 2014). Loss‐of‐function experiments in cell culture and mice demonstrated that Snail1 activity is indeed required for CAF activity. Inducible Snail1 depletion in established CAF lines decreases their paracrine activity on collective invasion of breast and colon tumour cells. Accordingly, epithelial tumour cells co‐xenografted with Snail1‐depleted fibroblasts originated tumours with lower invasion than those transplanted with control fibroblasts (Alba‐Castellón *et al*., 2016). Altogether, these data (summarized in Table 1) demonstrate that Snail1 activity is required for the activity of CAFs in promoting tumour progression.

**Table 1:** Summary of reported EMT‐TFs expressed in CAFs and their effects

Factors	Tumour type/Cells	Observations	Reference
Snail1	Cervical squamous cell carcinoma Colon carcinoma	Expression in tumour–stroma interface	Francí *et al*. (2006)
Snail1	Breast tumour	Expression in stroma	Côme *et al*. (2006)
Snail1	Colon carcinoma	Prognostic marker for stage I and II tumours.	Francí *et al*. (2009)
Snail1	Pharyngeal squamous cell carcinoma	Expression in stroma and endothelial cells predicts poor survival	Jouppila‐Mättö *et al*. (2011b)
Snail1‐Twist1	Pharyngeal squamous cell carcinoma	Expression in stroma predicts disease progression, while absence predicts better outcome	Jouppila‐Mättö *et al*. (2011a)
Snail1	Breast cancer Cultured colorectal CAFs	Prognostic marker in early‐infiltrating tumours Extracellular architecture control	Stanisavljevic *et al*. (2014)
Snail1	Colon carcinoma Cultured colorectal CAFs	Association with α‐SMA and FAP expression Specific cytokine profile	Herrera *et al*. (2014)
Snail1	Cultured colorectal CAFs Genetic breast cancer in mice	Secretion of diffusible signalling molecules promoting tumour invasion	Alba‐Castellón *et al*. (2016)
Twist1	Gastric carcinoma Cultured lung and skin fibroblasts	Coexpression with CAF markers Association with poor prognosis (diffuse‐type tumours)	Sung *et al*. (2011)
Twist1	Cultured human gastric fibroblasts and CAFs Gastric carcinoma	Activation by tumoral IL6 via p‐STAT3 Necessary for CAF activation CXCL12 Twist1 target that is associated with the presence of CAFs	Lee *et al*. (2015)
Twist1	Mammary xenografts	Downstream effector of CD44 activated CAFs	Spaeth *et al*. (2013)
Twist1/2	Colorectal cancer	Stromal Twist1/2 expression in budding colorectal tumours Twist1 associates with adverse features	Galván *et al*. (2015)
Twist1	Colorectal cancer Human fibroblast	Expression in tumour‐stroma Matrix stiffness control via paladin and ColA1	García‐Palmero *et al*. (2016)
ZEB1	Pancreatic ductal adenocarcinoma	Independent predictor of survival after resection	Bronsert *et al*. (2014)
ZEB1/2‐Snail1	Pancreatic ductal adenocarcinoma	Expression in tumour and stromal cells Only stromal ZEB2 significantly associated with metastasis	Galván *et al*. (2015)
ZEB2	Pharyngeal squamous cell carcinoma	More relapse for stroma‐positive tumours Better disease‐specific and overall survival for negative tumours	Jouppila‐Mättö *et al*. (2015)

As mentioned, Snail1 is expressed in myofibroblast implicated in wound healing (Francí *et al*., 2006). Indeed, induced depletion of Snail1 prevents the anisotropic organization of granulation tissue and delays wound healing (Stanisavljevic *et al*., 2014). Moreover, the factor is detected in conditions causing hyperstimulation of fibroblasts, such as fibromatosis, as well as in sarcomas and fibrosarcomas (Francí *et al*., 2006). Alba‐Castellón *et al*. showed that genetic depletion of Snail1 in mesenchymal stem cells (MSCs) deficient for the p53 tumour suppressor downregulates MSC markers and prevents these cells from producing sarcomas in immunodeficient SCID mice. Moreover, human sarcomas display high Snail1 expression, particularly in undifferentiated tumours, and this is associated with poor outcome (Alba‐Castellón *et al*., 2014).

Actions of Snail1 are also linked to cardiac myofibroblasts. First, it was found upregulated in the infarcted heart (Liu *et al*., 2012), and recently, Snail1 expression in cardiac fibroblasts has been confirmed to be required for deposition of collagen I, expression of fibrosis‐related genes and adoption of a myofibroblast fate. Published data suggest that Snail1 expression is induced in the cardiac fibroblasts after hypoxic injury and that it contributes to myofibroblast phenotype and fibrotic scar formation (Biswas and Longmore, 2016).

Following a hepatic injury that produces fibrosis, Snail1 was found to be a critical mediator of hepatic stellate cell activation to produce myofibroblasts (Scarpa *et al*., 2011). Goyal *et al*. correlated Snail1 expression with cutaneous fibrotic disorders. They found that Snail1 expression was low in normal skin but high in all fibrotic conditions studied, including skin biopsy specimens of keloid, hypertrophic scar, scleroderma, and nephrogenic systemic fibrosis (Goyal *et al*., 2016).

Therefore, collectively, all these data indicate that Snail1 expression controls the activity of most of the physiologically and pathologically activated fibroblasts, including CAFs.

### 2.2 Molecular mechanisms up‐ and downstream of fibroblastic Snail1

Basal expression of Snail1 in cultures of adult fibroblasts does not reflect its undetectable levels in adult tissue but likely reflects an artefactual expression in response to the culture conditions; however, even in these conditions, Snail1 is increased by TGF‐β (Batlle *et al*., 2013) and PDGF‐BB (platelet‐derived growth factor‐BB) through the phosphoinositide‐3‐kinase pathway (Rowe *et al*., 2009). Although no direct data on Snail2 expression or its potential role in CAFs and other activated fibroblasts have been reported, it is likely to play a role as, as for Snail1, its RNA levels increase in mouse embryonic fibroblasts activated with TGF‐β (Liu *et al*., 2013). TGF‐β has been described as a regulator of CAFs (Calon *et al*., 2014) and is also required for fibroblast activation in wound healing and fibrosis (Rybinski *et al*., 2014), pointing to TGF‐β as an effective paracrine inducer of Snail1 expression in adult fibroblasts.

On the other hand, mechanical signals regulate and activate Snail1 protein in CAFs. Zhang *et al*. described a sequence of events initiated by the induction of ROCK activity in response to ECM stiffness that indirectly stabilizes Snail1 protein by increasing intracellular tension, integrin clustering, and integrin signalling to ERK2. Increased ERK2 activity leads to nuclear accumulation of Snail1, and thus to avoidance of cytosolic proteasomal degradation (Zhang *et al*., 2016). This mechanism, which increases in tumour fibrosis, can perpetuate activation of CAFs to sustain tumour fibrosis and to promote tumour metastasis through the regulation of Snail1 protein level and activity (Baulida and García de Herreros, 2015; Zhang *et al*., 2016).

In contrast to the abundant literature characterizing Snail1‐dependent molecular mechanisms in EMT, only a few reports have addressed these mechanisms in fibroblast activation. Using cultured fibroblast activated with serum within a tissue‐like three‐dimensional extracellular matrix, Snail1 was suggested to have functions following terminal differentiation of mesenchymal cells. Specifically, Snail1‐deficient fibroblasts exhibit global alterations in gene expression, which include defects in invasive activity that depend on membrane‐type 1 matrix metalloproteinase (Rowe *et al*., 2009).

Snail1 was described to be required for the rapid TGF‐β‐induced RhoA activity upregulation that controls cytoskeletal rearrangements in fibroblasts (Stanisavljevic *et al*., 2014), although the detailed mechanism is still unaddressed. Two other reports indicate that Snail1 is required for driving a CAF‐specific secretome, including for MCP‐3 expression (Herrera *et al*., 2014) or for prostaglandin E2 (PGE2) secretion, likely through transcriptional control of PGE2 metabolism regulatory molecules (Alba‐Castellón *et al*., 2016). Snail1 also influences the level and activity of the mechanotransductor YAP1 in CAFs exposed to a stiff matrix (Zhang *et al*., 2016). Under such condition, Snail1/2 can form complexes with YAP/TAZ that control skeletal stem/stromal cell homeostasis and osteogenesis (Tang *et al*., 2016).

## 3 Twist protein expression in CAFs

During mouse embryogenesis, Twist1 is expressed in various mesodermal tissues (Füchtbauer, 1995; Stoetzel *et al*., 1995), but after birth, it is barely detectable in normal mesenchymal cells of adult tissues and limited to mesenchymal stem cells (Isenmann *et al*., 2009; Wang *et al*., 1997) and human white adipocytes (Pettersson *et al*., 2010). Twist1 overexpression has been reported in a variety of epithelial cancer cells with clinical correlations with poor prognosis, an event associated with its capacity to promote EMT (Ansieau *et al*., 2010). Recently, Twist1‐expressing stromal fibroblasts within cancer tissue have been observed (Table 1), but elucidating the clinical significance of this and the underlying regulating mechanism require more investigation (see below).

Using a Twist1 monoclonal antibody to analyse gastric cancer fibroblasts, researchers in Kim′s laboratory observed Twist1 expression in CAFs more frequently than in other cancer cells but rarely observed Twist1 to be expressed in noncancerous tissue (Sung *et al*., 2011). By laser capture microdissection, they detected that the Twist1 immunopositive stromal fibroblasts express significantly increased CAF markers, such as for the fibroblast‐specific protein 1 and CXCL14. Twist1 mRNA expression showed a significant linear correlation with that of PDGF receptors β and α. Conditioned media from Twist1‐expressing skin and lung fibroblasts significantly promote invasion of gastric cancer cells *in vitro* (Sung *et al*., 2011). In 195 gastric cancer samples, Twist1 expression in CAFs was associated with tumour size, invasion depth and lymph node metastasis. Twist1 was also associated with poor prognosis in patients with gastric cancer, particularly in those with the diffuse type (Sung *et al*., 2011). Later, they further demonstrated that Twist1 is a key regulator of CAFs. They show that proinflammatory cytokine IL6 that is commonly expressed in tumours was sufficient to induce Twist1 expression in normal cultured fibroblasts and to transdifferentiate them into CAFs through STAT3 phosphorylation. Twist1 expression was necessary and sufficient for CAF activation, and silencing its expression in CAFs inhibited their tumour‐promoting properties. Finally, the authors defined the chemokine CXCL12 as a Twist1 transcriptional target, and expression of both proteins correlated with the presence of CAFs in gastric tumour specimens (Lee *et al*., 2015).

Studies carried out in other laboratories also support a role for Twist1 in CAFs. In the tumour microenvironment of mammary xenografts, mesenchymal CD44 expression contributes to the acquisition of an activated fibroblast phenotype via Twist1 activation (Spaeth *et al*., 2013). Laser capture microdissection of epithelium and stroma from low‐ and high‐grade budding colorectal tumours shows Twist1 and Twist2 expression to be essentially restricted to stromal cells. Twist1, but not Twist2, staining was associated with adverse features, including a worse overall survival time (Galván *et al*., 2015). Similarly, in colorectal cancers, Twist1 expression was found to be mainly restricted to tumour–stroma. In this study, the authors show that Twist1‐induced activation of human fibroblasts promotes matrix stiffness by upregulating palladin and collagen α1(VI) (García‐Palmero *et al*., 2016).

More recently, an increased expression of fibroblastic Twist1 has been reported in fibrotic human and murine skin, which is dependent on TGF‐β/SMAD3. Twist1, in turn, enhances TGF‐β‐induced fibroblast activation in a p38 MAP kinase‐dependent manner. The stimulatory effects of Twist1 on resident fibroblasts were mediated by Twist1 homodimers. This is because TGF‐β upregulates the expression not only of Twist1 but also of Twist1 competitors for E12/E47 and, as a consequence, the formation of Twist1 homodimers is favoured that of Twist1‐E12/E47 heterodimers. Mice with selective depletion of Twist1 in fibroblasts are therefore protected from experimental skin fibrosis in different murine models to a comparable degree as mice with a ubiquitous depletion of Twist1 (Palumbo‐Zerr *et al*., 2017). Thus, Twist1 can be considered as a profibrotic factor in systemic sclerosis and a key regulator of CAFs.

## 4 ZEB protein expression in CAFs

Bronsert *et al*. (2014) identified stromal ZEB1 expression as an independent predictor of survival after resection of pancreatic ductal adenocarcinoma. In 117 cases included in the study, they found that high ZEB1 expression in cancer cells and CAFs was associated with poor prognosis. While there was also a trend for poor prognosis with a lymph node ratio, multivariate analyses showed stromal ZEB1 expression grade to be the only independent factor of survival after resection. In a different report, ZEB1, ZEB2 and Snail1 were also detected in tumour and stromal cells of pancreatic ductal adenocarcinoma; however, when the authors analysed the association between stromal expression and lymph node metastasis, only ZEB2 expression was significantly associated with metastasis (Galván *et al*., 2015). In pharyngeal squamous cell carcinoma, tumours with positive stromal ZEB2 staining relapsed more often than those with negative tumours, and negative stromal ZEB2 immunoreactivity correlated significantly with better disease‐specific survival and overall survival (Jouppila‐Mättö *et al*., 2015) (Table 1).

Further reports show ZEB1 and ZEB2 functions in nontumorigenic activated fibroblasts. Data from Chang *et al*. (2014) suggest that ZEB1 may participate in the pathogenesis of oral submucous fibrosis by activating the α‐SMA promoter and inducing myofibroblast transdifferentiation from buccal mucosal fibroblasts: silencing ZEB1 in fibrotic fibroblasts isolated from a patient suppressed the expression of α‐SMA and myofibroblast activity and inhibition of insulin‐like growth factor receptor‐1 could suppress ZEB1 activation in fibrosis. Zhou *et al*. (2015) studied post‐traumatic hypertrophic scars, a fibrotic disease with excessive ECM production by fibroblasts in response to tissue injury, and they demonstrated that the TGF‐β/miR200b/ZEB1 pathway might participate in this pathogenesis. Cunnington *et al*. found ZEB2 to be related to the myofibroblast phenotype. Specifically, they showed that ZEB2 protein expression increases in the infarct scar and propose that the Ski‐ZEB2‐Meox2 pathway provides a novel mechanism for regulation of the cardiac myofibroblast phenotype (Cunnington *et al*., 2014).

## 5 CAFs expressing EMT‐TFs prime EMT‐TF expression in tumour cells

As described above, EMT‐TF expression in CAF has been associated with stromal signatures and tumour malignance. In addition, some reports link the presence of a malignant stromal signature to the expression of EMT‐TFs in tumour epithelial cells. Thus, in ER‐positive and hormone‐treated breast cancer patients, Roman‐Pérez *et al*. describe an active subtype of breast microenvironment (with high expression of fibrosis and cell motility genes) strongly associated not only with lower overall survival and a TGF‐β‐induced fibroblast signature, but also with an epithelial Twist1 overexpression signature. The tumour tissue exhibited a higher density of Twist1 nuclear staining predominantly in epithelium (Román‐Pérez *et al*., 2012). In colon tumours, comparative RNA analyses in stroma^high^ (aggressive) and stroma^low^ tumours show that the neoplastic cells from stroma^high^ tumours express specific EMT drivers (ZEB2 and Twist1‐2) and that 98% of differentially expressed genes in these tumour types are strongly correlated with these two EMT‐TFs (Vellinga *et al*., 2016).

**Figure 1:**
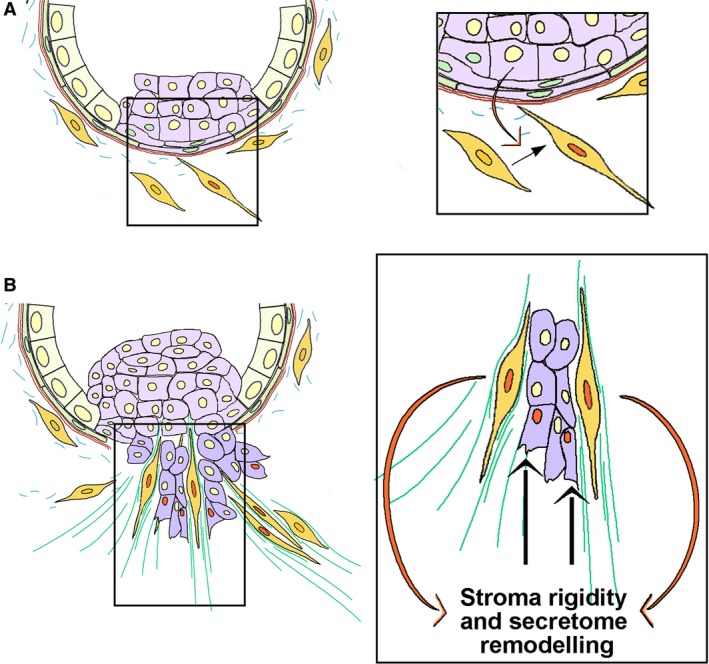
Paracrine signalling and mechanical signalling generated by CAFs in an EMT‐TF‐dependent manner promote the expression of EMT‐TFs in cancer epithelial cells. (A) Tissue homeostasis in mammary glands is maintained by a coordinated signalling between epithelial cells (yellow) and stromal cells (orange). Oncogenic mutations in epithelial cells fuel uncontrolled epithelial growth, generating primary tumour foci (purple cells). Paracrine signalling from tumour cells (orange arrow in the magnified box) promotes the expression of nuclear EMT‐TFs in adjacent fibroblasts (red nuclei). Additional colour coding: luminal cells, yellow nuclei; basal epithelial cells, green nuclei; normal fibroblasts, orange nuclei; basal lamina; red lines; extracellular fibres, blue lines. (B) In metastatic tumours, basal lamina is hampered and tumour cells escape from primary foci (dark purple cells). These events are facilitated by desmoplasia, microenvironmental changes promoted by CAFs expressing EMT‐TFs (orange cells with red nuclei), including fibrillar architecture and secretome remodelling (orange arrows and text in the amplified box). Signalling generated by local extracellular changes (black arrows) induces the expression of EMT‐TFs in adjacent tumour cells (dark violet cells with red nuclei), providing them with properties related to cancer malignance, such as stemness, increased tumour cell motility and chemoresistance. Therefore, EMT‐TFs induce changes in cell behaviour in both parenchymal and stromal tumour cells that support poor cancer prognosis.

Immunohistochemical analyses suggest that CAFs expressing EMT‐TFs promote expression of these factors in adjacent tumour cells. In a cohort of 162 colorectal tumours, 128 specimens expressed Snail1; of these 128, 96 were positive simultaneously for stroma and tumour cell, 24 were only stroma positive, and 4 expressed Snail1 exclusively in tumour cells (Francí *et al*., 2009). These data, together with the observation that stromal and epithelial Snail1 staining were in the same tumour area, suggest that the expression of Snail1 in the two compartments is somehow linked and that stromal expansion precedes tumour expression (Fig. 1). Similar observations were reported for the ZEB1 protein in a study of 117 pancreatic ductal adenocarcinomas. While all specimens displayed some degree of ZEB1 expression in the stroma, 63 did not express ZEB1 in the epithelial tumour cells, and high levels of stromal ZEB1 correlated with positive staining in the tumour (Bronsert *et al*., 2014).

A cascade of paracrine secreted factors regulated by EMT‐TFs might drive sequential expression of EMT‐TFs in stromal and parenchymal tumour cells but experimental evidence is still missing. In gastric cancers, IL6 activates fibroblastic Twist1, and the resulting CAFs secrete the chemokine CXCL12 in a Twist1‐dependent manner (Lee *et al*., 2015). Although CXCL12 promotes the expression of Snail1 (Liao *et al*., 2016; Lv *et al*., 2015) and Twist1 (Yao *et al*., 2016) in glioblastoma cancer cells, and of Snail1 in human oral squamous cell carcinomas (Taki *et al*., 2008), no reports show CXCL12‐induced expression of Snail1 or Twist1 in tumour cells of gastric cancers. However, CAFs expressing EMT‐TFs can also promote the expression of EMT‐TFs in adjacent epithelial cells by increasing the stroma rigidity (Fig. 1B). In breast tumours, the presence of dense clusters of collagen fibrils indicates increased matrix stiffness and correlates with poor survival (Conklin *et al*., 2011; Provenzano *et al*., 2008). It has been reported that Snail1‐expressing CAFs regulate ECM stiffness and that the presence of CAFs expressing Snail1 in early‐infiltrating breast tumours correlates with a tumour perpendicular fibre organization within the tumour and bad prognosis (Stanisavljevic *et al*., 2014). As mentioned above, rigid substrates stabilize nuclear Snail1 in breast tumour cells but also can drive EMT and tumour metastasis, through a Twist1‐G3BP2 mechanotransduction pathway. Specifically, high matrix stiffness promotes nuclear translocation of Twist1 by releasing it from its cytoplasmic binding partner G3BP2. In human breast tumours, collagen fibre alignment and reduced expression of G3BP2 together predict poor survival (Wei *et al*., 2015). Thus, by increasing stromal rigidity, CAFs can condition cancer cells to express EMT‐TFs even when tumour cells accumulate loss‐of‐function mutations in the signalling pathways that promote the EMT, such as the TGF‐β pathway, as described in some colorectal cancers (Fearon, 2011).

Based on these considerations, sequential EMT‐TF expression in tumour–stroma and parenchyma cells is envisioned to support tumour malignance (Fig. 1). Oncogenic mutations in epithelial cells initiate uncontrolled growth that generates primary tumour foci. Then, specific paracrine signalling secreted by tumour cells or cells activated by these tumour cells switch on the expression of EMT‐TFs in adjacent fibroblasts, which drives CAF activation (Fig. 1A). EMT‐TF‐dependent CAF activity on extracellular architecture and secretome promotes tumour malignance because microenvironmental changes stimulate EMT‐TFs expression in tumour cells that sustains stemness, increased tumour cell motility, and chemoresistance (Fig. 1B). Therefore, EMT‐TFs detection in parenchyma and stroma is indicative of coordinated changes in cell behaviour and poor cancer prognosis.

## 6 How does the activity of EMT‐TFs trigger and establish a CAF phenotype?

**Figure 2:**
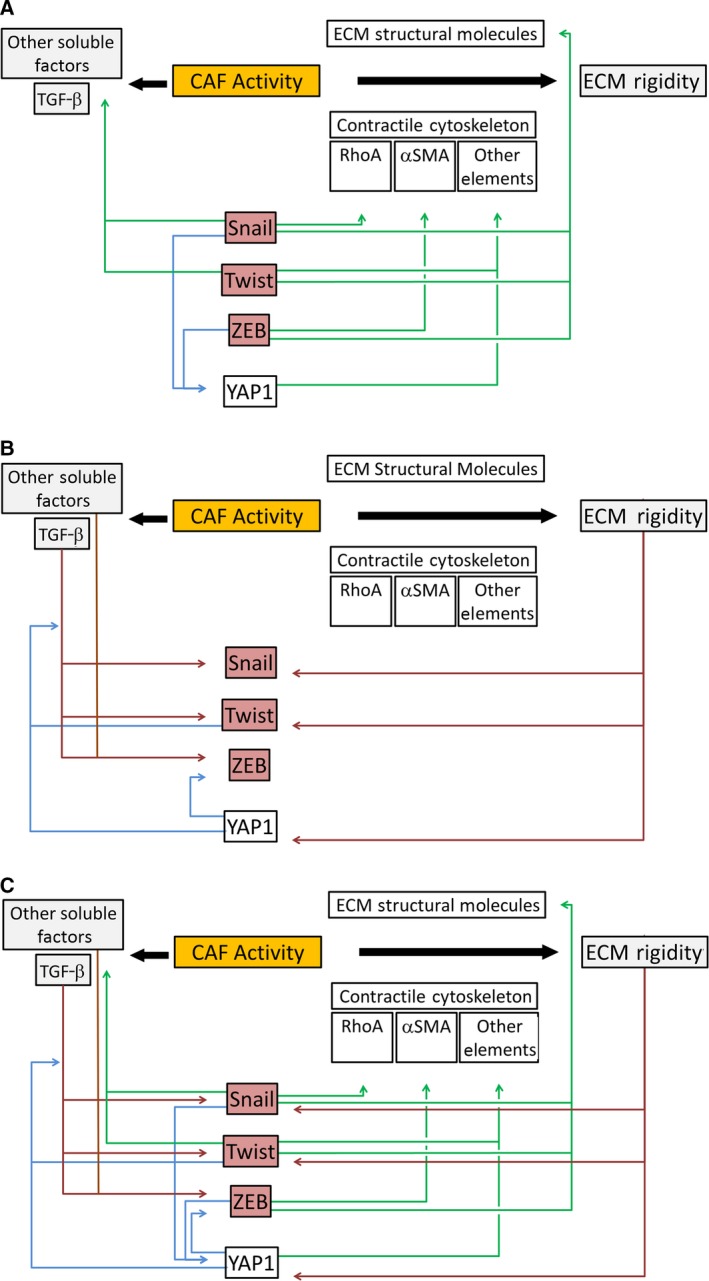
Putative EMT‐TF‐dependent mechanisms supporting CAF phenotype**.** The reported activities of EMT‐TFs in CAFs and other cells allow elucidation of EMT‐TF‐dependent mechanisms controlling CAF phenotype. (A) Putative EMT‐TF downstream mechanisms driving CAF activation are schematized. Snail1 and ZEB1 facilitate assembly and activity of the contractile cytoskeleton by modulating RhoA activity and αSMA transcription, respectively. Both proteins can interact with and modulate YAP1 activity, which controls regulatory molecules of the CAF cytoskeleton, such as ANLN, DIAPH3, and MYL9. Twist1 acts on the cytoskeletal protein palladin. On the other hand, extracellular molecules such as fibronectins, collagens, crosslinking enzymes or metalloproteases are targeted by the Snail, ZEB or Twist proteins. Through these pathways, EMT‐TFs regulate the CAF cytoskeleton assembly and activity and modify extracellular mechanics. Snail1 and Twist1 are also implicated in the secretion of soluble factors by CAFs, such as CXCL12, MCP‐3 and PGE2. (B) EMT‐TFs are likely to be central regulators of feed‐forward molecular loops that set the CAF phenotype. Twist1 induces an autoactivatory loop in TGF‐β signalling that prolongs the action of the cytokine on fibroblasts. Snail1, YAP1 and Twist1 are activated by extracellular rigidity generated by CAFs, allowing a mechanosensitive feed‐forward regulation of the CAF phenotype. YAP1 can also fuel TGF‐β‐induced EMT‐TF activity and favours ZEB1‐dependent transcription. Interactions between Snail1/2 and ZEB1 with YAP1 can further potentiate their activity. (C) The in/out and out/in EMT‐TF‐dependent mechanisms described in (A) and (B) that collectively sustain the CAF phenotype are represented.

The above‐summarized data point to signalling pathways regulated by EMT‐TFs that promote the CAF phenotype. However, detailed expression and cross‐regulation of the EMT‐TFs during the activation and stabilization of CAFs have yet to be characterized, and other yet‐undescribed EMT‐TF activities could be also required (i.e. pertaining to the stability or subcellular localization of regulatory proteins). To overcome these limitations, and to envision a sequence of events that could induce the CAF phenotype (Fig. 2), we can use reported EMT‐TF‐activities from other cell types to complement the known EMT‐TF actions in CAFs.

An initial expression of EMT‐TFs in fibroblasts can modulate the contractile actomyosin cytoskeleton at different regulatory points, thereby switching on the myofibroblast transition: (a) Snail1 triggers RhoA and the downstream effectors that assemble and activate the highly contractile α‐SMA actin fibres; (b) ZEB1 affects these fibres and induces myofibroblast transdifferentiation by controlling the α‐SMA promoter in oral submucous fibrosis; (c) Twist1 upregulates the cytoskeletal protein palladin; and (d) because Snail1/2 (Tang *et al*., 2016) and ZEB1 (Selth *et al*., 2017) influence the activity of YAP1 in several contexts, they could also modulate the YAP1 pathway in CAFs, thus modulating the extracellular matrix by increasing the expression of several cytoskeletal regulators (including ANLN and DIAPH3) and by controlling the protein levels of MYL9 (Calvo *et al*., 2013). In this way, EMT‐TFs can both directly and indirectly affect polymerization and remodelling of the extracellular fibres through focal adhesion‐transmitted tension (Fig. 2A,C).

As reviewed above, EMT‐TFs also affect the extracellular environment by increasing the expression of structural and soluble ECM molecules and the activity of remodelling enzymes. In activated fibroblasts, Snail1 induces the expression of fibronectin, collagen and the collagen‐crosslinking enzyme LOX1, which promote matrix rigidity. Similarly, Twist1 promotes collagen α1 expression. These data can be complemented by observations in cancer cells, in which ZEB1 induces LOXL2‐mediated collagen stabilization and deposition in the extracellular matrix (Peng *et al*., 2017), and Snail1 and Twist1 expression affects the activity of metalloproteases on the extracellular compartment (Ota *et al*., 2009; Weiss *et al*., 2012; Zhao *et al*., 2011) (Fig. 2A,C).

After its initial induction, the CAF phenotype can be stabilized by feed‐forward loops on EMT‐TFs. In fact, expression of Twist1 (Palumbo‐Zerr *et al*., 2017) and Snail1 (Baulida and García de Herreros, 2015; Zhang *et al*., 2016) in CAFs has been shown to induce autoactivatory loops on TGF‐β and mechanical signalling, respectively. Snail1 influences the level and activity of YAP1 in CAFs exposed to a stiff matrix generated by CAFs (Zhang *et al*., 2016), and YAP1 also mediates a feed‐forward regulation to fix the CAF phenotype. Reciprocally, YAP1 could modulate EMT‐TF activity, as described for endothelial cells in which YAP1 interacts with SMAD complexes and modulates TGF‐β‐induced upregulation of Snail1, Snail2 and Twist1 (Zhang *et al*., 2014a), and in breast cancers, in which ZEB1 turns into a transcriptional activator by interacting with YAP1 (Lehmann *et al*., 2016) (Fig. 2B,C).

In the multistep process of CAF activation, downregulation of p53 has been described to be relevant for downregulating the failsafe mechanism required for CAF hyperproliferation, causing the tumour‐associated fibrosis that is observed surrounding malignant epithelial tumours (Procopio *et al*., 2015). EMT‐TFs have been shown to modulate the p53 pathway in cancer cells: a Snail1/HDAC1/p53 trimolecular complex deacetylates active p53 and promotes its proteasomal degradation (Ni *et al*., 2016), and a Twist1/p53 complex destabilizes the oncosuppressive protein by altering specific post‐translational modifications (Piccinin *et al*., 2012). Therefore, modulating the p53 pathway could be one way that EMT‐TFs support CAF differentiation. Moreover, Twist1 and ZEB1 could promote CAF hyperproliferation by blocking cell cycle control through repression of cyclin‐dependent kinase inhibitors, as described for tumour cells (Ansieau *et al*., 2008; Burns *et al*., 2013; Ohashi *et al*., 2010).

Besides hyperproliferation, the action of EMT‐TFs on the p53 pathway can provide chemoresistance to CAFs, an event that likely promotes CAF survival and subsequent CAF‐facilitated tumour resistance during anticancer therapies. This could be the case for CAFs from pancreatic cancers, which have been shown to be intrinsically resistant to the chemotherapeutic gemcitabine (Richards *et al*., 2017). EMT‐TFs may also confer resistance to CAFs through other pathways described in cancer cells, as reviewed in detail elsewhere (Ansieau *et al*., 2014); such mechanisms include the action of ZEB1 on recombination‐dependent DNA repair through ubiquitin‐specific peptidase 7 (USP7) interaction and checkpoint kinase1 (CHK1) stabilization (Zhang *et al*., 2014b), Twist1 modulation of pro‐ and antiapoptotic members of the BCL‐2 family through AKT2 expression (Ansieau *et al*., 2010), and the action of Snail1 and Snail2 on radiation‐induced apoptosis by repressing PTEN phosphatase (Escriva *et al*., 2008) and Puma (Wu *et al*., 2005).

Although we cannot discard that some functional populations of CAFs – likely devoid of myofibroblastic and chemoprotective abilities – do not express EMT‐TFs, the levels of EMT‐TFs sustained by feedback mechanisms is a source of CAF heterogeneity. Anticancer therapies thus inadvertently are likely to select the antiapoptotic population of CAFs expressing EMT‐TFs over a threshold amount. These CAFs provide cancers with therapy resistance by mechanisms including the induction of EMT‐TF expression in tumour cells. Therefore, the exhaustive characterization of the EMT‐TF activity in CAFs is a promising research objective for discovering new targetable pathways that allow the current anticancer therapies to be improved.
